# ^1^H, ^13^C and ^15^N assignment of stem-loop SL1 from the 5'-UTR of SARS-CoV-2

**DOI:** 10.1007/s12104-021-10047-2

**Published:** 2021-08-28

**Authors:** Christian Richter, Katharina F. Hohmann, Sabrina Toews, Daniel Mathieu, Nadide Altincekic, Jasleen Kaur Bains, Oliver Binas, Betül Ceylan, Elke Duchardt-Ferner, Jan Ferner, Boris Fürtig, J. Tassilo Grün, Martin Hengesbach, Daniel Hymon, Hendrik R. A. Jonker, Bozana Knezic, Sophie M. Korn, Tom Landgraf, Frank Löhr, Stephen A. Peter, Dennis J. Pyper, Nusrat S. Qureshi, Andreas Schlundt, Robbin Schnieders, Elke Stirnal, Alexey Sudakov, Jennifer Vögele, Julia E. Weigand, Julia Wirmer-Bartoschek, Kerstin Witt, Jens Wöhnert, Harald Schwalbe, Anna Wacker

**Affiliations:** 1 Institute for Organic Chemistry and Chemical Biology, Goethe-University Frankfurt, Max-von-Laue- Straße 7, 60438 Frankfurt, Germany; 2 Center for Biomolecular Magnetic Resonance (BMRZ), Goethe-University Frankfurt, Max-von-Laue- Straße 7, 60438 Frankfurt, Germany; 3 Bruker BioSpin, Silberstreifen 4, 76287 Rheinstetten, Germany; 4 Present Address: BioNTech SE, An der Goldgrube 12, 55131 Mainz, Germany; 5 Institute for Molecular Biosciences, Goethe-University Frankfurt, Max-von-Laue-Straße 9, 60438 Frankfurt, Germany; 6 Present Address: Faculty of Chemistry, Weizmann Institute of Science, 7610001 Rehovot, Israel; 7 Institute for Biophysical Chemistry, Goethe-University Frankfurt, Max-von-Laue-Straße 9, 60438 Frankfurt, Germany; 8 Department of Biology, Technical University of Darmstadt, Schnittspahnstraße 10, 64287 Darmstadt, Germany; 9 Present Address: EMBL Heidelberg, Meyerhofstraße 1, 69117 Heidelberg, Germany; 10 Present Address: Deutero GmbH, Am Ring 29, 56288 Kastellaun, Germany

**Keywords:** SARS-CoV-2, 5'-UTR, SL1, Solution NMR spectroscopy, COVID19-NMR

## Abstract

The stem-loop (SL1) is the 5'-terminal structural element within the single-stranded SARS-CoV-2 RNA genome. It is formed by nucleotides 7–33 and consists of two short helical segments interrupted by an asymmetric internal loop. This architecture is conserved among Betacoronaviruses. SL1 is present in genomic SARS-CoV-2 RNA as well as in all subgenomic mRNA species produced by the virus during replication, thus representing a ubiquitous *cis*-regulatory RNA with potential functions at all stages of the viral life cycle. We present here the ^1^H, ^13^C and ^15^N chemical shift assignment of the 29 nucleotides-RNA construct 5_SL1, which denotes the native 27mer SL1 stabilized by an additional terminal G-C base-pair.

**Supplementary Information:**

The online version contains supplementary material available at 10.1007/s12104-021-10047-2.

## Biological context

**Fig. 1:**
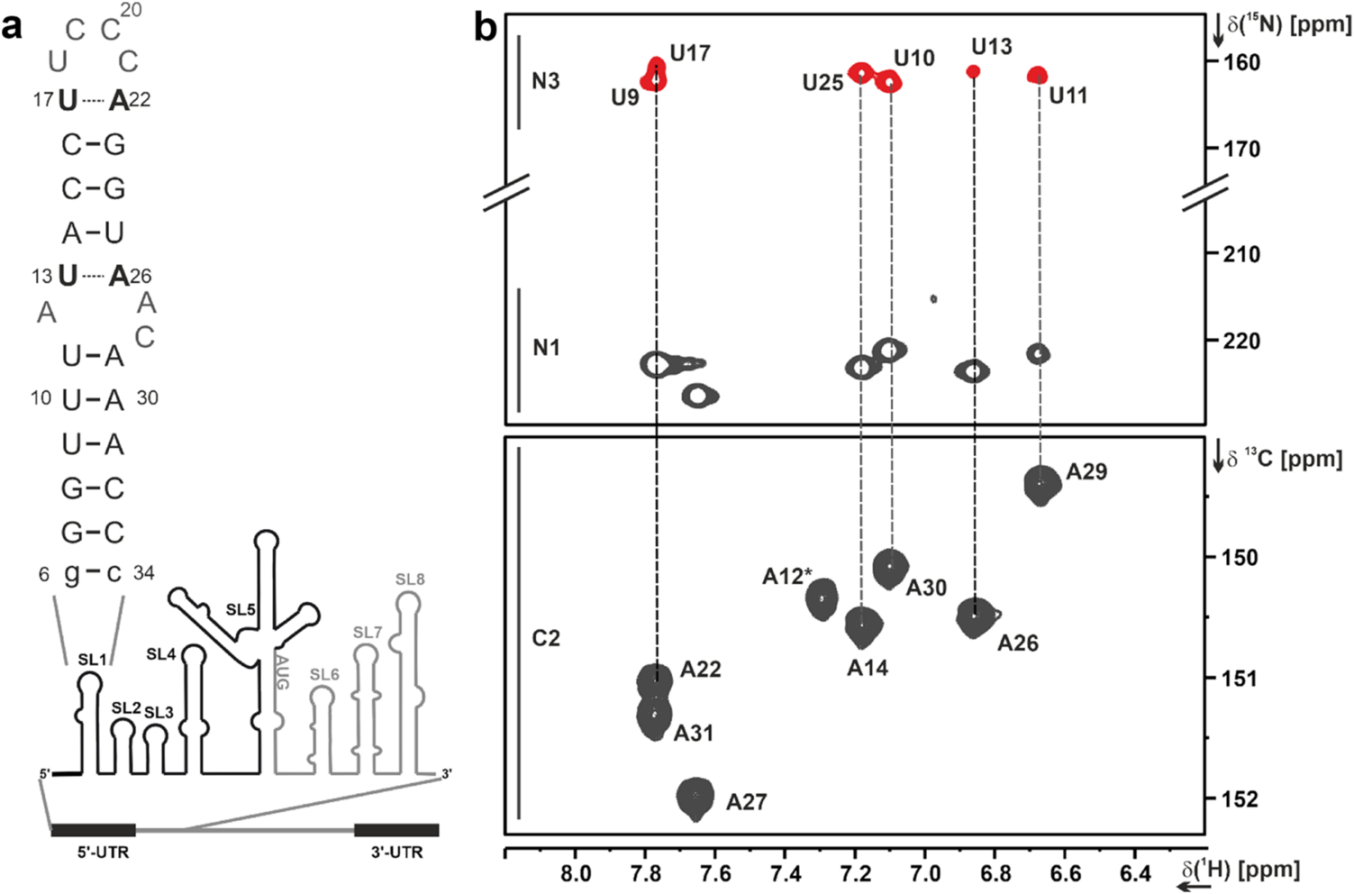
**a** Secondary structure of 5_SL1 and its genomic position within the 5'-UTR of the SARS-CoV-2 genome. **b** Detection of the W–C base-pairs U13-A26 and U17-A22 in the lrHNN-COSY experiment (Table 1, XIII.). Adenosine C2H2 resonances (lower spectrum, ^1^H,^13^C-HSQC) were used to assign the ^2^J-N1H2 diagonal peaks and the corresponding uridine N3 cross peaks. Note that the A12 N1H2 resonance is broadened beyond detection. The U13-A22 and U17-A22 correlations are shown in black, the other base-pairs in grey in panel **a**

The 5'-untranslated regions (5'-UTR) of Betacoronavirus RNA genomes contain several highly conserved, structured RNA elements that play essential roles in viral RNA synthesis. SL1, the first of these RNA stem-loops, has been structurally characterized by NMR spectroscopy in Mouse hepatitis virus (MHV), Bovine coronavirus (BCoV), and the human coronavirus HCoV-OC43 (Liu et al. 2007). Despite local differences in RNA sequences, the ~ 37 nucleotides (nt) stem-loop adopts a very similar secondary structure in all three viruses, consisting of two helical parts interrupted by a stretch of nucleotides with mismatched bases and capped by a less conserved apical loop. Extensive mutational studies of MHV SL1 accompanied by NMR showed that virus viability depends on the sequence of the lower part of SL1 and on the stability of the upper part of SL1 (Li et al. 2008). For SL1 from SARS-CoV, it was shown that it can replace MHV SL1 and restore virus replication (Kang et al. 2006), suggesting a functionally equivalent role for SL1 in Betacoronaviruses in general. Subsequently, for the human pathogenic viruses MERS-CoV, SARS-CoV, and SARS-CoV-2, an additional function for SL1 was described. Here, SL1 is involved in viral escape from non-structural protein 1-mediated translational shutdown (Tanaka et al. 2012; Terada et al. 2017; Tidu et al. 2020). At present, the predicted secondary structure of stem-loop SL1 in SARS-CoV-2 (Fig. 1) has been experimentally verified (Miao et al. 2020; Wacker et al. 2020; Iserman et al. 2020; Manfredonia et al. 2020). SL1 is formed by nucleotides 7–33 of the 5'-UTR. The 5-base-pair (bp) lower helix is separated from the 3-bp upper helix by an asymmetric 5-nt internal loop flanked on both sides by A–U Watson–Crick (W–C) base-pairs. The UUCCCA apical loop has been mapped as an interaction site with the host protein LARP1 (Schmidt et al. 2020).

## Methods and NMR experiments

RNAs were synthesized by *in vitro* run-off transcription from linearized DNA plasmids as previously described (Wacker et al. 2020; Schnieders et al. 2021; Vögele et al. 2021). For DNA template production, the sequence of SL1 (RNA sequence 5'gGGUUUAUACCUUCCCAGGUAACAAACCc-3') together with the T7 promoter was generated by hybridization of complementary oligonucleotides and introduced into the EcoRI and NcoI sites of an HDV ribozyme (Schürer et al. 2002) encoding plasmid, based on the pSP64 vector (Promega). RNAs were transcribed as a fusion construct with the 3'-HDV ribozyme to obtain homogeneous 3RNAs were transcribed as a fusion construct with the 3'-HDV ribozyme to obtain homogeneous 3'-ends. Transformation and amplification of the recombinant vector pHDV-5_SL1 was done in the *Escherichia coli* strain DH5α. Plasmid-DNA was purified using a large scale DNA isolation kit (Gigaprep; Qiagen) according to the manufacturer’s instructions and linearized with HindIII prior to *in vitro* transcription with T7 RNA polymerase [P266L mutant, prepared as described in (Guilleres et al. 2005)]. RNA amounts sufficient for NMR experiments were produced in 15 ml preparative transcription reactions [20 mM dithiothreitol, 2 mM spermidine, 200 ng/µl plasmid template, 200 mM Tris/glutamate (pH 8.1), 30 mM Mg(OAc)_2_, 12 mM rNTPs, 32 µg/ml (^15^N,^13^C-labelled RNAs)/150 µg/ml (uniformly ^15^N labelled RNA) T7 RNA Polymerase]. After 1 h incubation time, yeast inorganic phosphatase [9.6 µg/mL (^15^N,^13^C-labelled RNAs)/4.8 µg/mL (uniformly ^15^N labelled RNA) final concentration] was added. Transcription reactions (6 h at 37 °C and 70 rpm) were terminated by addition of EDTA (80 mM final concentration) and NaOAc (0.3 M final concentration). After transcription, RNAs were precipitated by adding 1 volume equivalent of ice-cold 2-propanol and incubation for 1 h at − 20 °C. For purification, RNA fragments were separated on 12 % denaturing polyacrylamide (PAA) gels and visualized by UV shadowing at 254 nm. SL1 RNAs were excised from the gel and incubated at − 80 °C for 30 min, followed by 15 min at 65 °C in 0.3 M NaOAc. Elution was achieved overnight by passive diffusion into 30 mL 0.3 M NaOAc solution. RNAs were precipitated by addition of 4 volume equivalents of ethanol at − 20 °C overnight. If the absorption ratio 220/260 nm of the RNA after dissolving in water was higher than 1.5, RNA was desalted via PD10 columns (GE Healthcare) for the following HPLC. Residual PAA was removed by reversed-phase HPLC using a Kromasil RP-18 column and a gradient of 0–40 % 0.1 M acetonitrile/triethylammonium acetate. After freeze-drying of RNA-containing fractions and cation exchange by LiClO_4_ precipitation [2 % (w/v) in acetone], the RNA was folded in water by heating to 80 °C followed by rapid cooling on ice. Buffer exchange to NMR buffer (25 mM potassium phosphate buffer, pH 6.2, 50 mM potassium chloride) was performed using Vivaspin centrifugal concentrators (2 kDa molecular weight cut-off, Sarstedt). Purity of SL1 was verified by denaturing PAA gel electrophoresis and homogenous folding was monitored by native PAA gel electrophoresis, loading the same RNA concentration as used in NMR experiments (Fig. S1).

Using this protocol, four NMR samples of 5_SL1 were prepared and used for the assignment presented herein: A 0.64 mM uniformly ^15^N labelled RNA sample and a 1.2 mM uniformly ^15^N,^13^C-labelled RNA sample, each in NMR buffer with 5 % (v/v) D_2_O for a 5 mm Shigemi tube and 7 % (v/v) D_2_O for a 1.7 mm NMR tube, a 1.33 mM uniformly ^15^N,^13^C-labelled RNA in 99.95 % (v/v) D_2_O and an 0.87 mM selectively ^15^N,^13^C(A/C)-labelled RNA in NMR buffer (5 % (v/v) D_2_O).

**Table 1:** List of NMR experiments, “(Bruker)” indicates the NMR experiments that were carried out at Bruker BioSpin, Rheinstetten

NMR experiment	Experimental parameters
**I. 2D** ^**1**^**H**,^**1**^**H** **NOESY** **A**: (Bruker) aromatics, in 99.95 % D_2_O **B**: Iminos and aromatics with excitation sculpting (Hwang and Shaka 1995; Sklenar 1995)	A: 800 MHz, 298 K, ns: 16, sw(f2): 12.0 ppm, sw(f1): 6.5 ppm, aq(f2): 319 ms, aq(f1): 162 ms, o1(^1^H): 4.7 ppm, o2(^13^C): 118 ppm, o3(^15^N): 190 ppm, rel. delay: 1.5 s, NOE mixing time: 150 and 300 ms, time: 14 h B: 900 MHz, 283 K, ns: 64, sw(f2): 22.2 ppm, sw(f1): 11.8 ppm, aq(f2): 102 ms, aq(f1): 45 ms, o1(^1^H): 4.7 ppm, o2(^13^C): 110 ppm, o3(^15^N): 153 ppm, rel. delay: 1.45 s, NOE mixing time: 80 , 160 and 240 ms, time: 29 h
**II. 2D** ^**1**^**H**,^**13**^**C-HSQC** **A**: Aromatics **B**: C1′-H1′ (Bodenhausen and Ruben 1980), optimized in-house	A: 700 MHz, 298 K, ns: 4, rel. delay: 1.0 s, sw(f2): 9.2 ppm, sw(f1): 10 ppm, aq(f2): 67 ms, aq(f1): 85 ms, o1(^1^H): 4.7 ppm, o2(^13^C): 142.5 ppm, o3(^15^N): 153 ppm, INEPT transfer time: 2.7 ms, off-resonant Q3 shaped pulse for C5 decoupling at 95 ppm with 25 ppm bandwidth, time: 35 min B: 600 MHz, 298 K, ns: 4, rel. delay: 1.0 s, sw(f2): 8.7 ppm, sw(f1): 22.7 ppm, aq(f2): 84 ms, aq(f1): 32 ms, o1(^1^H): 4.7 ppm, o2(^13^C): 90.5 ppm, o3(^15^N): 154 ppm, INEPT transfer time: 2.9 ms, off-resonant Q3 shaped pulse for C2′ decoupling at 72 ppm with 12 ppm bandwidth, time: 20 min
**III. 2D** ^**1**^**H**,^**13**^**C-ct-HSQC** All CH, optimized for ribose resonances (Vuister and Bax 1992)	700 MHz, 298 K, ns: 32, sw(f2): 8.3 ppm, sw(f1): 105 ppm, aq(f2): 102 ms, aq(f1): 16 ms, o1(^1^H): 4.7 ppm, o2(^13^C): 105 ppm, rel. delay: 1.0 s, INEPT transfer time 2.9 ms, constant-time period: 25 ms, time: 5 h
**IV. 3D TROSY-(H)CCH-COSY** Adenine base sin systems (Simon et al. 2001)	950 MHz, 298 K, ns: 8, sw(f3, ^1^H): 9.0 ppm, sw(f2, ^13^C): 26.2 ppm, sw(f1, ^13^C): 58.1 ppm, aq(f3): 119 ms, aq(f2): 5.1 ms, aq(f1): 4.6 ms, o1(^1^H): 4.7 ppm, o2(^13^C): 142.5 ppm, o3(^15^N): 150 ppm, rel. delay: 1.0 s, time: 21 h
**V. 2D BEST-TROSY-H(N)CO** (Favier and Brutscher 2011; Solyom et al. 2013)	600 MHz, 283 K, ns: 128, sw(f2): 21.0 ppm, sw(f1): 31 ppm, aq(f2): 63 ms, aq(f1): 13,6 ms, o1(^1^H): 4.7 ppm, o2(^13^C): 157 ppm, o3(^15^N): 153 ppm, rel. delay: 0.3 s, HN-INEPT transfer time: 5.2 ms, NC-INEPT transfer time 18 ms, time: 1.5 h
**VI. 2D** ^**13**^**C-detected** ^**1**^** C**,^**15**^**N-HSQC** C2/4/6 to Amino-N2/4/6′ (Bermel et al. 2006; Fiala and Sklenár 2007)	800 MHz, 298 K, ns: 32, rel. delay: 2.5 s, sw(f2, ^13^C): 50 ppm, sw(f1, ^15^N): 43 ppm, aq(f2): 51 ms, aq(f1): 16 ms, o1(^13^C): 160 ppm, o2(^15^N): 86.5 ppm, INEPT CN transfer time: 18 ms, time: 2.5 h
**VII. 3D HCN** (Bruker) H6/8/H1′-to-N9/N1, in 99.95 % D_2_O (Fiala et al. 1998)	800 MHz, 298 K, ns: 8, sw(f3, ^1^H): 8.9 ppm, sw(f2, ^13^C): 28 ppm, sw(f1, ^15^N): 31 ppm, aq(f3): 143 ms, aq(f2): 8.5 ms, aq(f1): 32 ms, o1(^1^H): 4.7 ppm, o2(^13^C): 113.5 ppm, o3(^15^N): 157 ppm, rel. delay: 1.0 s, INEPT HC transfer time: 2.8 ms, INEPT CN transfer time: 30 ms, time: 1 d 15 h
**VIII. 3D** ^**13**^**C-detected (H)CNC** C1′-to-C6/8 Modified from Fiala et al. (1998)	800 MHz, 298 K,, ns: 24, sw(f3, ^13^C): 24 ppm, sw(f2, ^15^N): 34 ppm, sw(f1, ^13^C): 12 ppm, aq(f3): 67 ms, aq(f2): 23 ms, aq(f1): 25 ms, o1(^13^C): 90 ppm, o2(^1^H): 7.6 ppm, o3(^15^N): 157 ppm, rel. delay: 0.5 s, C6/8-N1/9 transfer time 30 ms, C–H transfer time 2.9 ms (1′) and 2.6 ms (6/8), time: 2 d 10 h
**IX. 3D (H)CCH TOCSY** **A**: C1′ to C2′; **B**: C1′ to C5′ (Kay et al. 1993; Richter et al. 2010)	700 MHz, 298 K, ns: 16, sw(f3,^1^H): 10.4 ppm, sw(f2,^13^C): 10.0 ppm, sw(f1,^13^C): 35.4 ppm, aq(f3): 82 ms, aq(f2): 26 ms, aq(f1): 12 ms, o1(^1^H): 4.7 ppm, o2(^13^C): 39 ppm, o3(^31^P): − 1 ppm, rel. delay: 1.0 s, CC-TOCSY mixing time (dipsi3 spin-lock): A: 6 ms, B: 18 ms, time: 2 d 2 h
**X. 3D FW-directed H(C)CH-TOCSY** (Schwalbe et al. 1995; Glaser et al. 1996)	700 MHz, 298 K, ns: 8, sw(f3,^1^H): 8.3 ppm, sw(f2,^13^C): 38.5 ppm, sw(f1,^1^H): 4.1 ppm, aq(f3): 87 ms, aq(f2): 8 ms, aq(f1): 27 ms, o1(^1^H): 4.7 ppm, o2(^13^C): 77 ppm, o3(^15^N): 155 ppm, rel. delay: 1.0 s, constant-time period: 8.3ms; CC-TOCSY mixing time (dipsi3 spin-lock): 9.2 ms, time: 1 d 22 h
**XI.** ^**13**^**C-NOESY-HSQC** **A**: 3D (Bruker) in 99.95 % D_2_O; **B**: 2D, sel. ^13^ C,^15^ N(A,C)-labelled RNA (Sklenář et al. 1993; Piotto et al. 1992)	800 MHz, 298 K, A (constant time in t2): ns: 8, sw(f3,^1^H): 12 ppm, sw(f2,^13^C): 105 ppm, sw(f1,^1^H): 5.9 ppm, aq(f3): 106 ms, aq(f2): 23 ms, aq(f1): 17 ms, o1(^1^H): 4.7 ppm, o2(^13^C): 108.5 ppm, o3(^15^N): 105 ppm, rel. delay: 1.0 s, HC-INEPT transfer time: 3 ms, constant-time period: 8.8 ms, NOE mixing time: 150 ms, time: 1 d 19 h B: ns: 64, sw(f2): 8.8 ppm, sw(f1,^1^H): 6.2 ppm, aq(f2): 73 ms, aq(f1): 51 ms, o1(^1^H): 4.7 ppm, o2(^13^C): 144 ppm, o3(^15^N): 154 ppm, rel. delay: 0.9 s, HC-INEPT transfer time: 2.8 ms NOE mixing time: 200 ms, time: 11 h
**XII. 2D** ^**13**^**C**,^**15**^**N(F2)-filtered NOESY** All-to-G/U protons (Ogura et al. 1996; Zwahlen et al. 1997; Breeze 2000; Iwahara et al. 2001)	900 MHz, 298 K, ns: 48, sw(f2): 12 ppm, sw(f1,^1^H): 9 ppm, aq(f2): 94 ms, aq(f1): 51 ms, o1(^1^H): 4.7 ppm, o2(^13^C): 120 ppm, o3(^15^N): 117 ppm, rel. delay: 1.5 s, NOE mixing time: 150 ms, time: 14 h
**XIII. 2D** ^**1**^**H**,^**15**^**N-BEST-TROSY-lrHNN-COSY** (Sklenár et al. 1994; Hennig and Williamson 2000; Farjon et al. 2009; Dingley and Grzesiek 1998; Dingley et al. 2008)	600 MHz, 298 K, ns: 512, sw(f2): 9.8 ppm, sw(f1): 88.9 ppm, aq(f2): 87 ms, aq(f1): 14.8 ms, o1(^1^H): 7 ppm, o2(^13^C): 150 ppm, o3(^15^N): 192 ppm, rel. delay: 0.3 s, HN-INEPT transfer time: 19 ms, NN-transfer time 22.5 ms, time: 11 h
**XIV.** ^**1**^**H**,^**1**^**H-TOCSY** (Shaka et al. 1988; Hwang and Shaka 1995)	700 MHz, 283 K, ns: 16, sw(f2): 8.8 ppm, sw(f1): 6.2 ppm, aq(f2): 100 ms, aq(f1): 51 ms, o1(^1^H): 4.7 ppm, o2(^13^C): 101 ppm, o3(^15^N): 85 ppm, rel. delay: 1.0 s, TOCSY mixing time (dipsi3 spin-lock): 30 ms, time: 3 h

## Assignment strategy and extent of assignment

Based on our previously reported assignment of the base-paired imino groups, the amino groups of base-paired cytidines and the adenosine H2 protons for 5_SL1 (Wacker et al. 2020), we have already confirmed the overall secondary structure of 5_SL1 consisting of two helical regions. For the stably base-paired adenosine and cytidine residues, we have previously also reported the assignments of the hydrogen bond-acceptor nitrogens in the HNN-COSY experiment.

Starting from these available assignments and following the classical NOE-based strategy, we first assigned all anomeric H1′ protons and all aromatic H6 (pyrimidine)/H8 (purine) protons via one single “sequential walk” in a 2D NOESY spectrum acquired in D_2_O (Table 1, I.). For the nucleotides U9/U10, U18/C19, and C20/C21, the anomeric-aromatic walk was ambiguous in the H1′–H6/8-region due to severe signal overlap. However, these connectivities could be unambiguously established via the intra-nucleotide and sequential H2′_*i*_–H8/H6_*i, (i−1)*_ NOEs. Within the H1′–H6/H8 region of the NOESY, also the pyrimidine (intraresidual) H5–H6 and adenosine H1′_*i*_–H2_*(i+1) intra−strand, (i+1) cross−strand)*_ NOE signals are typically observed. The 2D NOESY experiment, in combination with a 2D ^1^H,^1^H-TOCSY experiment showing only the pyrimidine H5–H6 cross peaks, thus allowed the unambiguous assignment of all pyrimidine H5 and adenosine H2 protons. All protonated nucleobase carbons as well as the C1’ carbons were assigned in ^1^H,^13^C-HSQCs optimized for the respective CH-transfer (Table 1, II. and III.). Correlations from purine C8H8 and adenosine C2H2 resonances were used as starting points to assign all adenosine and guanosine N7/N9 resonances and adenosine N1/N3 resonances in the 2D ^1^H,^15^N-^2J^HSQC as described in (Wacker et al. 2020), except for the A12 N1 resonance, which was not observable, most likely due to exchange broadening. For the adenosines, all base ^13^C nuclei were assigned by correlating the C2H2 and C8H8 resonances with the quaternary base carbons C4, C5, and C6 in the 3D TROSY-(H)CCH-COSY experiment (Table 1, IV.). The same experiment also yielded assignments for guanosine C4 and C5 resonances. Uridine C2/C4 and guanosine C2/C6 resonances were assigned by correlating the respective imino protons to the carbonyl resonances in a 2D H(N)CO experiment (Table 1, V.). ^15^N resonances of all exocyclic adenosine amino groups were identified in a ^13^C-detected 2D ^13^C,^15^N-HSQC (Table 1, VI.). Ribose spin systems were connected to their respective nucleobases by simultaneously correlating C1’ and C6 (for pyrimidine nucleobases) or C8 (for purine nucleobases) to the glycosidic (N1/N9) nitrogen atom in ^1^H-detected 3D HCN and ^13^C-detected 3D (H)CNC experiments (Table 1, VII. and VIII.), verifying the sequential NOE-based assignment of the H1′ protons. 3D (H)CCH-TOCSY experiments were used to identify the carbon resonances of the ribose spin systems. Discrimination of C2′ and C3′ was achieved by varying the CC-TOCSY mixing time to either correlate C1′and C2′ during a short TOCSY mixing time (6 ms) or to correlate C1′ to all ribose carbons via a long TOCSY mixing time of 18 ms (Table 1, IX). Due to severe resonance overlap of the respective C1′H1′ resonances, the carbon spin systems for G6, G7, and G24 were not unambiguously resolved. In summary, about 90 % of the ribose H2′–H5′/H5″ resonances were assigned via a 3D forward-directed HCCH-TOCSY experiment (Table 1, X.), a 3D ^13^C-NOESY-HSQC (Table 1, XI.) and 2D ^13^C-filtered/edited NOESY experiments (Table 1, X. and XI.) on a selectively ^13^C,^15^N (A/C)-labelled sample.

## Internal loop

According to our previously reported secondary structure determination of 5_SL1, the internal loop consists of nucleotides A12-U13 and A26–A27-C28 (Wacker et al. 2020). A26 and A27 could both be potential interaction partners for U13, as observed for the homologous RNA element in MHV for A35 and A36 (Liu et al. 2007). However, formation of a W-C-type U13-A26 interaction was unambiguously observed in the lrHNN-COSY experiment (Table 1, XIII. and Fig. 1)), which in turn precluded a significantly populated U13-A27 interaction and eventually confined the internal loop to nucleotides A12, A27 and A28. The ^2^J_NN_ coupling for U13N3-A26N1 was 4.5 Hz as derived from the intensity ratio of cross peak to diagonal peak according to I_cross_/I_dia_ = – tan^2^(πJ_NN_τ) (Bax et al. 1994). For comparison, ^2^J_NN_ couplings for U11N3-A29N1, U10N3-A30N1, and U25N3-A14N1 were around 6.4 Hz, 6.6 Hz, and 6.7 Hz, respectively. The intraresidual N1 resonance of A12 was the only missing signal in the H2-N1/N3 correlation experiment, hinting at severe exchange-induced line-broadening. Note that this experiment clearly rules out disappearance of signals due to solvent exchange.

**Fig. 2:**
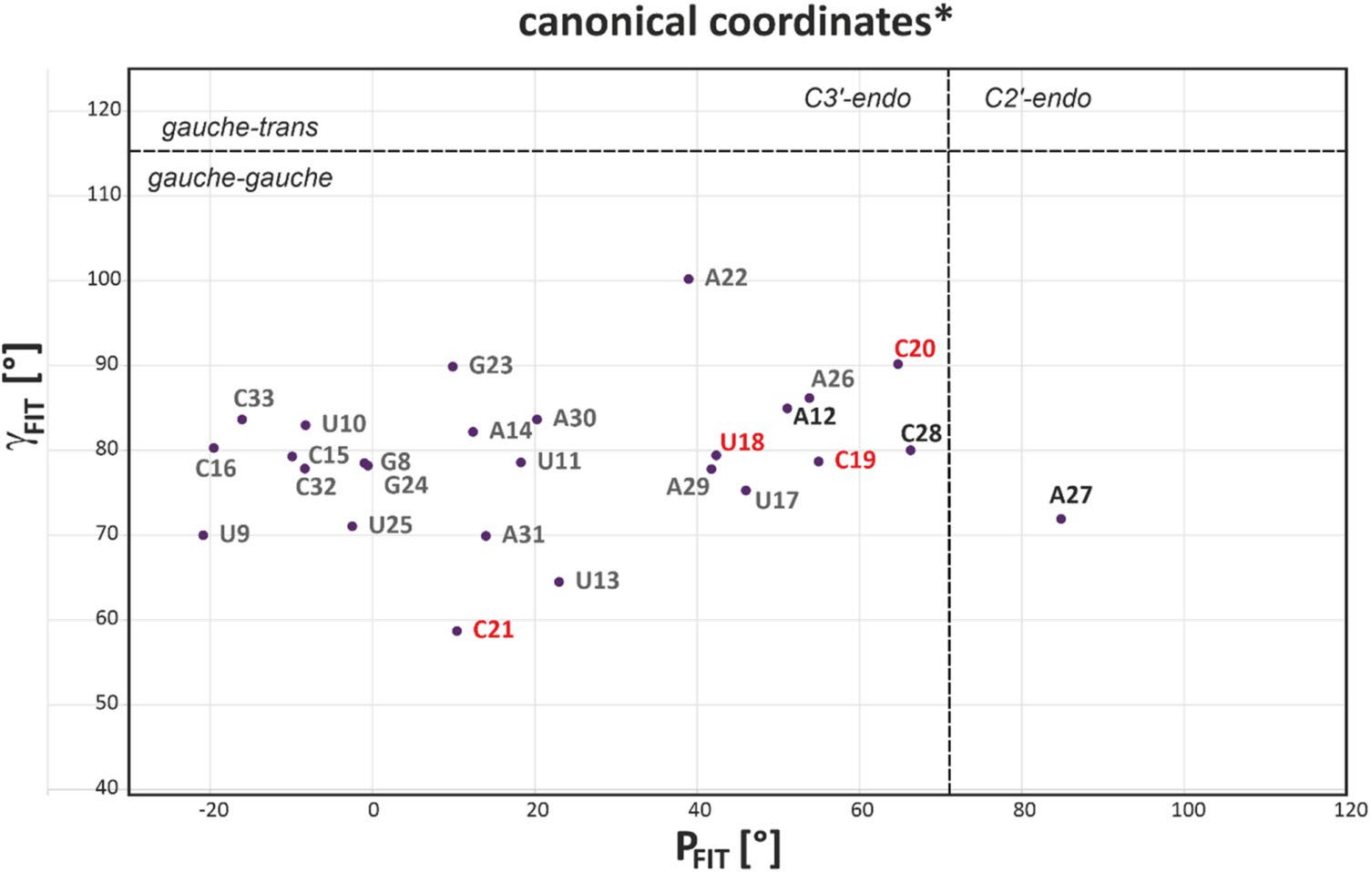
Plot of γ_FIT_ against P_FIT_ as calculated from ribose ^13^C chemical shifts according to (Cherepanov et al. 2010). Residues from the apical loop are marked in red, bulge residues in black. C34 is omitted due to its low-field C2′ chemical shift typical for the 3'-terminal nucleotide, resulting in exceptionally high values of the canonical coordinates

Empirical determination of ribose conformation by means of the canonical coordinates yielded no significant deviation from A-form helical structure for A12 and C28 (Fig. 2), whereas A27 was found to adopt a C2′-endo conformation. Qualitative evaluation of glycosidic torsion angles via the intensity of the intra-base H1′–H6/H8 NOESY cross peak did not reveal a tendency for *syn* conformation for any of the internal loop nucleotides. Furthermore, global chemical shift analysis using CS-Annotate (Zhang et al. 2021) supported a largely stacked arrangement of all nucleobases of the internal loop, except for C28 (SI Fig. S2).

## Pyrimidine loop

**Fig. 3:**
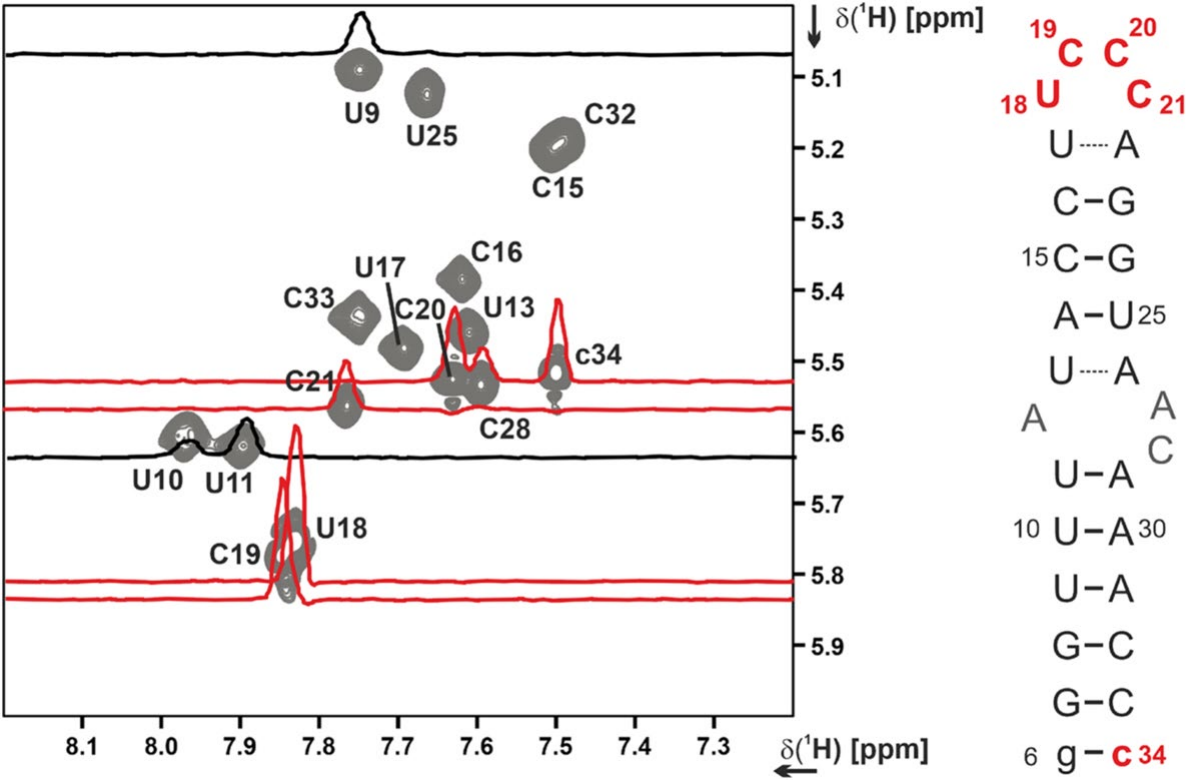
Expanded region of the 2D ^1^H,^1^H TOCSY experiment (Table 1, XIV.) correlating pyrimidine H5–H6 proton chemical shifts via their ^3^J coupling. Linewidths are approximately inversely proportional to the base order parameter, resulting in sharp signals for flexible residues that exhibit a lower than the global τ_c_. 1D traces for selected residues are shown in the 2D. The flexible loop residues U18, C19, and C20 and the non-native 3'-terminal c34 are highlighted in red; helical residues U9 and U11 are shown in black

The apical loop of 5_SL1 is formed by nucleotides U17-A22. For U17-A22, formation of a labile W–C base-pair was observed in the lrHNN-COSY (Fig. 1). Overlap of the A22 and A27 N1H2 resonances did not allow us to derive the ^2^J_NN_ coupling constant for A22N1-U17N3 in the same way as for the other A–U base-pairs as described above, but the U17N3 cross peak showed a reduced intensity compared to the canonical A–U base-pairs (Fig. 1). Ribose carbon chemical shifts of both nucleotides yielded canonical coordinates consistent with A-form conformation. Taken together, these results indicated that U17-A22 rather extends the upper helix by one base-pair, while the apical loop is a tetraloop formed by nucleotides U18 to C21. Linewidths in the TOCSY experiment were narrow for U18, C19, C20 and medium for C21, indicating conformational flexibility of this region (Fig. 3). The downfield chemical shifts of the U18 and C19 C6H6 groups were a further indication that these nucleotides are solvent-exposed and likely not participate in extensive stacking interactions. The Y-rich loop of 5_SL1 is currently discussed as a binding site for the Y-motif binding protein LARP1 (Schmidt et al. 2020). This protein-RNA interaction would severely impact the conformational flexibility of the involved nucleotides. Thus, the resonances of pyrimidines U18, C19, C20 and C21 may serve as valuable reporters for future structural investigations of RNA-protein interactions involving the apical loop of 5_SL1.

## Conclusions

It is common in NMR spectroscopy of RNA to consider W–C base-pairs as “stable” if the H-bonding imino proton is significantly protected from solvent-exchange and gives rise to an observable imino proton signal. Relying on the presence of imino proton signals only, the upper helix of SARS-CoV-2 5_SL1 consists only of three stable base-pairs, as these signals for U13 and U17 are missing even at 275 K. Available secondary structure predictions (Tavares et al. 2020; Rangan et al. 2020; Andrews et al. 2021), however, base pairs U13-A26 and U17-A22 are consistently present. We show here that these base pairs are at least significantly populated via the lrHNN-COSY experiment. This demonstrates the unique ability of solution NMR spectroscopy to capture subtle differences in secondary structure stability under given conditions. In SARS-CoV-2, the lower helix appears to be the most stable part of 5_SL1, which is in contradiction to the putative function in genome cyclization and the observed lability of the lower SL1 helices in MHV, HCoV-OC43, and BCoV (Li et al. 2008). Interestingly, long-range RNA-RNA interactions have been recently mapped for SARS-CoV-2 involving the 5'-UTR downstream elements SL2 and SL3 as interaction sites with the 3'-UTR (Ziv et al. 2020). Thus, the function of genome cyclization might have been handed over to other conserved RNA structures in SARS-CoV-2 while acquiring distinct functions for SL1 not yet described for its counterparts in MHV or BCoV. These functions may include protecting viral mRNA from translation shutdown (Tidu et al. 2020). Our extensive assignment of ^1^H, ^13^C and ^15^N chemical shifts for 5_SL1 provides experimental data as the basis for in-depth structural characterization of this stem-loop RNA and refines the currently available structure models in terms of structural dynamics, which is essential e.g., for the identification of potential drug binding sites.

## Data deposition

The BMRB deposition with the accession code 50349 was updated with the assignments reported herein.

## Supplementary Information

Below is the link to the electronic supplementary material.

Supplementary material 1 (DOCX 373.1 kb)
