# Supplementary material for: 1H, 13C and 15N assignment of stem-loop SL1 from the 5'-UTR of SARS-CoV-2
Source: Biomol NMR Assign. 2021 Aug 28;15(2):467–74. doi: 10.1007/s12104-021-10047-2 (PMC8401371; doi:10.1007/s12104-021-10047-2)
Supplement: Supplementary file 1 — Supplementary material 1 (DOCX 373.1 kb) [file 12104_2021_10047_MOESM1_ESM.docx]

**Supplementary Information to:**

**^1^H, ^13^C and ^15^N Assignment of Stem-Loop SL1 from the 5'-UTR of SARS-CoV-2**

Christian Richter^1,2^*, Katharina F. Hohmann^1,2^*, Sabrina Toews^1,2^, Daniel Mathieu^3^, Nadide Altincekic^1,2^, Jasleen Kaur Bains^1,2^, Oliver Binas^1,2,4^, Betül Ceylan^1,2^, Elke Duchardt-Ferner^2,5^, Jan Ferner^1,2^, Boris Fürtig^1,2^, J. Tassilo Grün^1,2,6^, Martin Hengesbach^1,2^, Daniel Hymon^1,2^, Hendrik R. A. Jonker^1,2^, Bozana Knezic^1,2^, Sophie M. Korn^2,5^, Tom Landgraf^1,2^, Frank Löhr^2,7^, Stephen A. Peter^8^, Dennis J. Pyper^1,2^, Nusrat S. Qureshi^1,2,9^, Andreas Schlundt^2,5^, Robbin Schnieders^1,2,10^, Elke Stirnal^1,2^, Alexey Sudakov^1,2^, Jennifer Vögele^2,5^, Julia E. Weigand^8^, Julia Wirmer-Bartoschek^1,2^, Kerstin Witt^1,2^, Jens Wöhnert^2,5^, Harald Schwalbe^1,2Ɨ^, Anna Wacker^1,2Ɨ^

* equal contribution


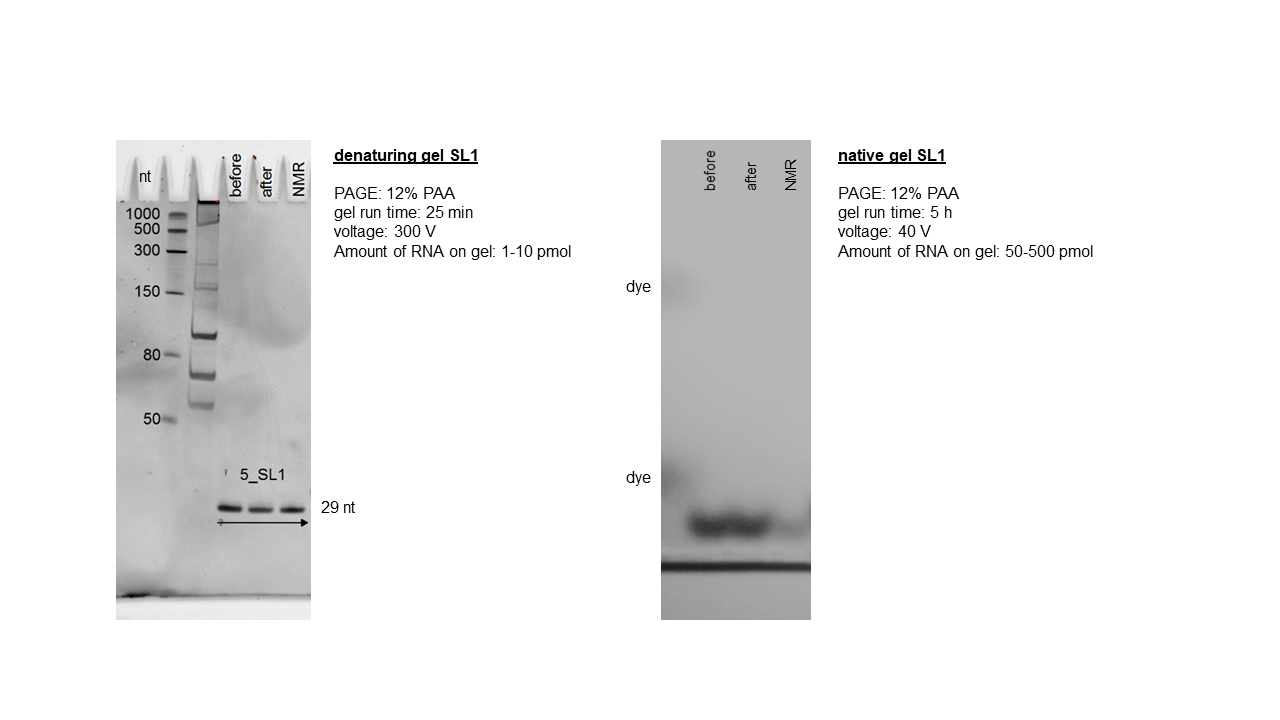
Supplementary figure S1: (left) Analytic denaturing PAA gel (12% PAA, Tris/borate/EDTA buffer) of SL1 in water before folding, after RNA folding (in water by heating to 80 °C followed by rapid cooling on ice) and the final NMR sample in NMR buffer. Visualization by GelRed and UV light. In all three cases, a pure RNA band is visible at expected position. (right) Native PAA gel (12% PAA, Tris/Acetate buffer, electrical power <1 W, water cooling) of SL1 before, after folding and the final NMR sample. Visualization by UV light. In all three cases, the gel confirms homogeneous, monomeric fold of the RNA.

Supplementary figure S2: Graphical output file of CS-Annotate (Zhang et al. 2021). The percentages of respective conformations are depicted as pie charts. For generation of the figure shown here, only ^13^C and ^1^H chemical shifts of CH and CH_2_ groups were used as input.

**Supplementary Literature**

Zhang K, Abdallah K, Ajmera P, et al (2021) CS-Annotate: A Tool for Using NMR Chemical Shifts to Annotate RNA Structure. J Chem Inf Model. https://doi.org/10.1021/acs.jcim.1c00006
